# The Effects of Endocrine Disrupting Chemicals on Biomarkers of Inflammation Produced by Lipopolysaccharide Stimulated RAW264.7 Macrophages

**DOI:** 10.3390/ijerph16162914

**Published:** 2019-08-14

**Authors:** Vedastus W. Makene, Edmund J. Pool

**Affiliations:** Department of Medical Bioscience, University of the Western Cape, Bellville 7535, South Africa

**Keywords:** Estradiol, 5α-dihydrotestosterone, Bisphenol A, anti-inflammatory, nitric oxide, interleukin 6

## Abstract

Endocrine disrupting chemicals (EDCs) are common pollutants in the environment and can induce disruption of the endocrine and immune systems. The present study evaluated the effects of selected common environmental EDCs on secretion of inflammatory biomarkers by RAW264.7 cells. The EDCs investigated were Estradiol (E2), 5α-dihydrotestosterone (DHT), and Bisphenol A (BPA). To evaluate if the effects caused by EDCs were modulated by steroid hormone receptors, antagonists of estrogen and androgen receptors were used. The steroid receptor antagonists used were Tamoxifen, an estrogen receptor antagonist, and Flutamide, an androgen receptor antagonist. Secretion of biomarkers of inflammation, namely nitric oxide (NO) and interleukin 6 (IL-6), were monitored. The NO was determined using Griess reaction and IL-6 was measured by enzyme linked immunosorbent assay (ELISA). Although 5 μg/mL E2, DHT, and BPA were not toxic to RAW264.7 cell cultures, the same treatments significantly (*p* < 0.001) reduced both NO and IL-6 secretion by lipopolysaccharide (LPS)-stimulated RAW264.7 cell cultures. The suppression of NO and IL-6 secretion indicate inhibition of inflammation by DHT, E2, and BPA. The inhibitory effects of DHT, E2 and BPA are partially mediated via their cellular receptors, because the effects were reversed by their respective receptor antagonists. Flutamide reversed the effects of DHT, while Tamoxifen reversed the effects of E2 and BPA. In conclusion, E2, BPA, and DHT inhibit the synthesis of inflammation biomarkers by LPS-stimulated RAW264.7 cells. The inhibitory effects of EDCs can be partially reversed by the addition of an estrogen receptor antagonist for E2 and BPA, and an androgenic receptor antagonist for DHT. The inhibition of inflammatory response in stimulated RAW264.7 cells may be a useful bioassay model for monitoring estrogenic and androgenic pollutants.

## 1. Introduction

Endocrine disrupting chemicals (EDCs) are environmental compounds that can interfere with biosynthesis, secretion, action, or metabolism of endogenous hormones, resulting in altered normal hormone actions [1,2]. They include compounds such as industrial chemicals, agricultural chemicals like pesticides, natural and synthetic hormones, and pharmaceuticals. These chemicals are widely distributed in the environment and especially in wastewater. Common examples reported in the environment are estrogenic steroids like Estradiol (E2) and Bisphenol A (BPA) [3,4] and androgenic steroids like testosterone and 5α-dihydrotestosterone (DHT) [5,6]. While BPA is an industrial chemical with many applications, testosterone and E2 are natural hormones of male and female animals, respectively.

Both estrogenic and androgenic chemicals are common pollutants in municipal wastewater. They are persistent in the environment and, in most cases, are resistant to removal by common conversional wastewater treatment techniques. Therefore, some of these pollutants can pass through wastewater treatment plants (WWTPs) and contaminate the environment. For example, Liu et al. [6] reported the presence of estrogenic E2 among several steroids in the environment after analysis of sludge and surface water samples. In another study, Sun et al. [3] reported some E2 and BPA activities in both sewage and reclaimed water.

Similar to estrogenic EDCs, androgenic steroids are also common in sewage and surface water bodies. Bell et al. [5] reported high androgenic activities in sewage samples. Along with other androgenic hormones, 5α-dihydrotestosterone (DHT) has also been reported in sewage [6]. DHT is a more biologically active form of testosterone. It is a derivative of testosterone synthesized from testosterone by enzymatic action of 5-reductase [7,8].

Exposure to environmental estrogenic and androgenic compounds causes many adverse health effects on the endocrine system, leading to several disorders in humans and animals [1,2,9,10]. The most common reported effects are reproductive disorders, including reproductive anomalies, sexual dysfunctions, and cancers of reproductive origin [11]. Apart from disruption of reproductive structure and functions, estrogenic and androgenic compounds are also known to induce immunotoxicity [12]. They induce immunotoxicity by interfering with activation and survival of immune cells; and alteration of cytokines and synthesis of inflammatory mediators [9]. The altered immune response is normally characterized by either suppression of immunity or stimulation of immune response. The immune suppression is normally characterized by enhanced disease susceptibility and inability to eliminate cancer cells [13,14]. On the other hand, the overstimulation of the immune system is characterized by immune hypersensitivity [15].

The inflammatory reaction is an important immunological response, which is induced by many inflammatory factors. These inflammatory factors include pathogens, injury, organic compounds, toxins, and endotoxins like lipopolysaccharide (LPS) [16]. The resulting inflammatory responses involve many immune cells. One of these types of cells is macrophages, which are phagocytic cells derived from circulating monocytes. They form the first line of the innate part of the immune system. When macrophages are stimulated, they ingest and digest invading pathogens, dead cells, and any other foreign substances [17]. Therefore, macrophage stimulation is one of the early immune responses to infection. For example, exposure of macrophages to LPS, a gram negative bacteria membrane component, induces an inflammatory response. The induction of an inflammatory response by LPS is through activation of a Toll-like receptor (TLR-4), which is expressed on the cell surface of macrophages [17,18].

Estrogenic and androgenic compounds can also induce an immune response in macrophages through their specific receptors, namely, estrogen receptors (ERs) and androgen receptors (ARs), respectively [19,20]. Both ERs and ARs are members of the nuclear receptor family of ligand-dependent transcription factors [21]. Therefore, exposure of macrophages to estrogenic and androgenic compounds may activate or inhibit expression of nuclear factor kappa B (NFκB), which leads to alteration of inflammatory mediators and cytokine secretion [18]. Inhibition of an inflammatory response induced by estrogen has been reported in wound healing [22]. On the other hand, suppression effects of testosterone on inflammation have been associated with many metabolic diseases [23]. In fact, recently, there is evidence in literature showing that a low testosterone level correlates with occurrence of metabolic diseases, characterized by increased expression of biomarkers of inflammation [24].

Activated macrophages initiate inflammatory responses by inducing secretion of many inflammatory mediators and pro-inflammatory cytokines [25]. Examples of common inflammatory mediators and pro-inflammatory cytokines secreted by macrophages are nitric oxide (NO) and interleukin 6 (IL-6), respectively [26]. IL-6 is a pro-inflammatory cytokine with many functions, including supporting chronic inflammatory reaction, induction of acute phase reactions, and inducible nitric oxide synthase (iNOS) [26,27]. Inducible nitric oxide synthase (iNOS) is a key enzyme in NO production. NO is a small molecule secreted by many tissue cells including macrophages. Therefore, secretion of both NO and IL-6 in macrophages is extensively used as a biomarker of inflammation studies [28,29,30].

Inflammatory responses have been studied in vitro using established cell lines like mouse RAW264.7 macrophages. This cell line was developed from mouse ascites leukemia cells. When RAW264.7 cells are stimulated, they secrete inflammatory mediators like NO and IL-6. Stimulation of RAW264.7 cells with LPS has been used extensively to study the immunomodulating effects of natural products and herbs [31,32,33,34]. However, there are relatively very few reports on the use of RAW264.7 cells to study anti-inflammatory effects of common pollutants like DHT, E2, and BPA. The present study was done to evaluate the in vitro effects of common EDCs, such as DHT, E2, and BPA, on cytotoxicity and inflammatory biomarker secretion by LPS-stimulated RAW264.7 macrophages. This study also evaluated whether effects of the EDCs on inflammatory biomarkers are mediated via their respective steroid receptors.

## 2. Material and Methods

### 2.1. Reagents

Lipopolysaccharides (LPS) from *Escherichia coli* 0111:B4, Dimethyl sulfoxide (DMSO), 5α-dihydrotestosterone (DHT), Estradiol (E_2_), Bisphenol A (BPA), Flutamide, and Tamoxifen were purchased from Sigma-Aldrich Chemie Gmbh, Munich, Germany. The LPS, DHT, E2, BPA, flutamide, and tamoxifen were dissolved in DMSO (Sigma-Aldrich, Germany).

### 2.2. Cells Culture

A mouse macrophage RAW264.7 cell line, purchased from American Type Culture Collection (ATCC TIB-71, Manassas, VA, USA), was used in this study. The RAW264.7 cells were cultured in Dulbecco’s Modified Eagle’s medium (DMEM, Lonza, Cape Town, South Africa), supplemented with 10% heat inactivated fetal bovine serum (FBS), 1% *v*/*v* antibiotic/antimycotic mixture (Sigma-Aldrich), 0.5% *v*/*v* gentamycin (Sigma-Aldrich), and 1% *v*/*v* glutamax (Gibco, Life Technology, Carlsbad, CA, USA). The cells were cultured in 96-well plates at a density of 5 × 10^5^ cells/mL in a humidified incubator at 37 °C and 5% CO_2_ until confluent. At confluence, cells were treated as follows: Normal medium for negative control and medium supplemented with 1 μg/mL lipopolysaccharides (LPS) from *Escherichia coli* 0111:B4 (Sigma-Aldrich, Germany) as a positive control. Some cell cultures stimulated with LPS (1 μg/mL) were treated with 5 μg/mL of each of DHT, E2, and BPA alone or in combination with 2 μg/mL of flutamide or tamoxifen. The optimal concentration of compounds was determined by serial dilution (data not shown). The dilution of DHT, E2, and BPA started with 1 in 100 of 10 μg/mL of compound. The concentrations of EDCs starting from 1 in 200 (5 μg/mL) and further dilutions did not affect cell viability and were not different from the control. Optimal concentrations of flutamide and tamoxifen treatment were also determined by serial dilution, starting from 1 in 500 dilutions of 5 μg/mL of each. The concentrations of flutamide and tamoxifen, starting from 1 in 1000 (2.5 μg/mL), and further dilutions alone had no suppression effects on cell viability, NO, and IL-6 secretion in RAW264.7 cells. The cells were cultured in medium supplemented with LPS (1 μg/mL) overnight. Then, cell cultures stimulated with LPS were treated with 5 μg/mL of each of DHT, E2, and BPA alone or in combination with 2 μg/mL of flutamide or tamoxifen and incubated at 37 °C and 5% CO_2_ overnight. After overnight incubation at 37 °C and 5% CO_2_, culture supernatants were collected for NO and IL-6 assays. The cells remaining on the plate were used for cell viability assays. Each assay was carried in four replicates.

### 2.3. Cell Viability

The cell viability was determined using the chromogenic WST-1 assay. The assay is based on the breakdown of the water-soluble (2-(4-Iodophenyl)-3-(4-nitrophenyl)-5-(2,4-disulfophenyl)-2H-tetrazolium by the dehydrogenase enzyme to produce water-soluble formazan dye that can be monitored spectrophotometrically. In brief, the assay procedure was as follows: After removal of cell supernatant from culture, each plate well received 100 μL of medium supplemented with 10% WST-1 reagent (Roche, Basel, Switzerland). The absorbance was read immediately after addition of WST-1 medium and a second reading was done after incubation for 30 min at 37 °C and 5% CO_2_. The change in absorbance at 450 nm over 30 min was used as a measure of cell viability.

### 2.4. Nitric Oxide Determination

Nitric oxide (NO) secreted by cells into the cell culture was determined using the Griess reaction. Cell culture supernatant in 96-well plates (Nunc, Roskilde, Denmark) was mixed with an equal volume of Griess reagent made up of 1% m/v sulphanilamide (Sigma-Aldrich, Germany), 0.01% m/v naphthyl ethylenediamine dihydrochloride (Sigma-Aldrich), and 2.5% phosphoric acid. The mixture was allowed to react for 15 min at room temperature. The colour developed was measured at 540 nm using a microplate reader (Multiskan Ex, Thermo Electron Corporation). The concentration of NO was determined from a standard curve generated using 1.56–100 μM sodium nitrite (Sigma-Aldrich, Germany).

### 2.5. Interleukin-6 Determination

Interleukin 6 (IL-6) in culture supernatant was determined using a double antibody sandwich enzyme linked assay (DAS ELISA), with a commercial kit (e-Bioscience, Ready-Set-Go, Waltham, MA, USA). The assays were done on Nunc Maxisorp 96-well plates (Nunc, Denmark). The ELISA kit contains all reagents required for the assay and the manufacturers’ assay protocol was followed. In brief, the protocol involved coating the ELISA plate with capture antibody, anti-mouse IL-6 diluted in coating buffer (PBS), and incubated overnight at 37 °C. Then, the plate was washed five times in wash buffer made of PBS with 0.1% *v*/*v* Tween. After washing, the plate was blocked with assay diluent for 1 h at room temperature. After another five washes, IL-6 standard and cell culture supernatant were added to each well accordingly and incubated for two hours at room temperature. The plate was washed again five times, then detection antibody, biotinylated anti-mouse IL-6, was added to each well and incubated for 1 h at room temperature. After another wash, Avidin—horse radish peroxidise (HRP) conjugate—was added and incubated for 30 min at room temperature. The plate was washed seven times, then TMB substrate was added and incubated in the dark for 15 min at room temperature. The reaction was stopped with 0.5M H_2_SO_4_ stop solution, and the absorbance was read at 450 nm with a multiskan microplate reader (Multiskan Ex, Thermo Electron Corporation).

### 2.6. Statistical Analysis

The data are presented as mean ± SD (*n* = 4), which were statistically analyzed with one way variance analysis (ANOVA) using sigmastat (SigmaStat software, Inc., CA, USA). The results of cell viability are presented as a percentage of the negative control. The mean values of NO and IL-6 concentration for each treatment were compared with the positive control. *p*-value <0.001 was considered statistically significant.

## 3. Results

### 3.1. Effects of Selected EDCs on Cell Viability

**Figure 1:**
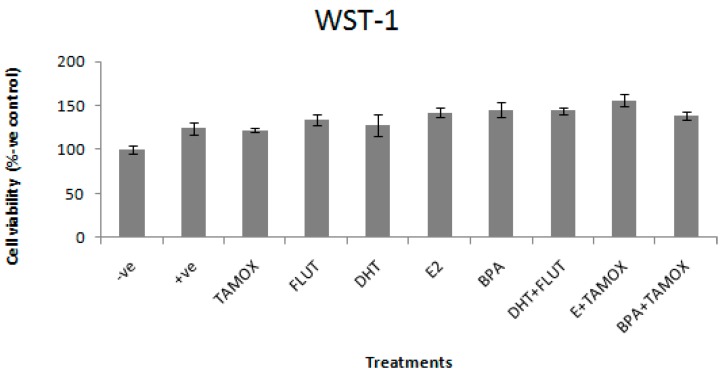
Effects of 5α-dihydrotestosterone (DHT), Estradiol (E2), and Bisphenol A (BPA) alone and in combination with flutamide or tamoxifen on viability of RAW264.7 cells, stimulated with 1 μg/mL lipopolysaccharide (LPS). The stimulated cells were treated with 5 μg/mL of DHT, E2, or BPA; and flutamide (FLUT) or tamoxifen (TAMOX) at 2 μg/mL; negative control was treated with normal medium and positive control was treated with LPS (1 μg/mL). The results of cell viability are presented as percentage of negative control.

The effects of DHT, E2, and BPA alone and in combination with flutamide or tamoxifen on viability of stimulated RAW264.7 cells are shown in Figure 1. The cell viability is presented as a percentage of the negative control. None of the treatments reduce RAW264.7 cell viability as compared with the positive control treated with LPS (1 μg/mL).

### 3.2. Effects of Selected EDCs on NO Production

**Figure 2:**
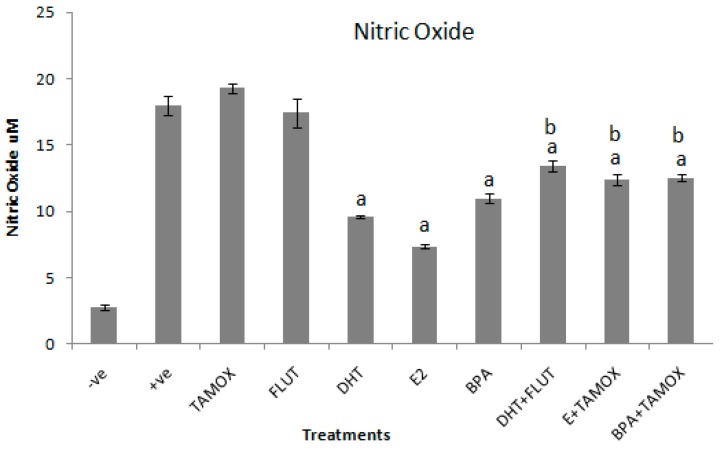
Effects of DHT, E2, and BPA alone and in combination with tamoxifen (TAMOX) or flutamide (FLUT) on secretion of nitric oxide (NO) in supernatant of RAW264.7 cells stimulated with 1 μg/mL LPS. The stimulated cells were treated with 5 μg/mL of DHT, E2, and BPA alone and 2 μg/mL of flutamide or tamoxifen. Negative control was treated with normal medium and positive control treated with LPS (1 μg/mL). The results are presented as mean ± SD; **a**-indicates that NO level is significantly (*p* < 0.001) lower than the positive control; **b**-indicates that NO level is significantly (*p* < 0.001) higher than levels of NO secreted by cells treated with DHT, E2, and BPA alone.

The effects of DHT, E2, and BPA alone and in combination with flutamide or tamoxifen on NO production in stimulated RAW264.7 cells were determined by NO assay in cell culture supernatant. The results of the effects of DHT, E2, and BPA alone and in combination with flutamide or tamoxifen on NO secretion in RAW264.7 cells stimulated with 1 μg/mL LPS are shown in Figure 2.

The results reveal that exposure of the stimulated cells to tamoxifen or flutamide alone had no significant effect on NO secretion. Exposure of the stimulated culture cells to 5 μg/mL of DHT, E2, and BPA significantly (*p* < 0.001) decreased NO secretion when compared to the positive control. The strongest suppression effect was induced by E2 (7.4 ± 0.13 μM). Next to E2 was DHT (9.6 ± 0.13 μM) and relatively weak suppression of NO production was induced by BPA (10.9 ± 0.4 μM). The inhibitory effects of EDCs were reversed by the addition of their respective antagonist compounds. Addition of flutamide, a chemical with anti-androgenic effects, significantly (*p* < 0.001) reversed the anti-inflammatory effects of DHT. The NO production induced by DHT alone was 9.6 ± 0.13 μM, whereas DHT in combination with flutamide induced 13.4 ± 0.13 μM of NO. Similarly, addition of tamoxifen, a chemical with anti-estrogenic effects, significantly (*p* < 0.001) reversed the effects of both estradiol and BPA. E2 alone induced 7.4 ± 0.13 μM, while E2 in combination with tamoxifen increased NO production to 12.4 ± 0.42 μM. BPA alone induced 10.9 ± 0.4 μM of NO. BPA in combination with tamoxifen induced 12.5 ± 0.28 μM of NO.

### 3.3. Effects of Selected EDCs on IL-6 Secretion

**Figure 3:**
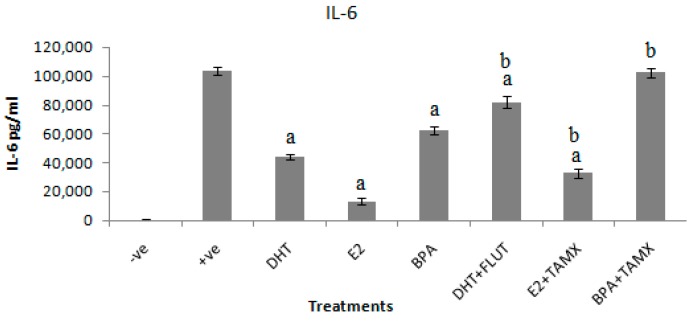
Effects of DHT, E2, and BPA alone and in combination with flutamide or tamoxifen on IL-6 secretion in RAW264.7 cells stimulated with 1 μg/mL LPS. The stimulated cells were treated with 5 μg/mL of DHT, E2, and BPA and 2 μg/mL of flutamide (FLUT) or tamoxifen (TAMOX). Negative control was treated with normal medium and positive was control-treated with LPS (1 μg/mL). The results are presented as mean ± SD; **a**-indicates that IL-6 level is significantly (*p* < 0.001) lower than the positive control; **b**-indicates that IL-6 level is significantly (*p* < 0.001) higher than levels of IL-6 secreted by cells treated with DHT, E2, and BPA alone.

The effects of DHT, E2, and BPA alone and in combination with flutamide or tamoxifen on secretion of IL-6 in stimulated RAW264.7 cells was determined in cell culture supernatant, using a double antibody sandwich enzyme linked assay (DAS ELISA). The effects of DHT, E2, and BPA alone and in combination with flutamide or tamoxifen on secretion of IL-6 in stimulated RAW264.7 cells are shown in Figure 3. The results show that exposure of cells to DHT, E2, and BPA alone significantly (*p* < 0.001) reduced the IL-6 response in stimulated RAW264.7 cells.

The results show that the positive control treated with LPS (1 μg/mL) secreted IL-6 as high as 103,914 ± 2620 pg/mL. Addition of DHT alone to stimulated cultures suppressed IL-6 secretion to 43,943 ± 1750 pg/mL. DHT in combination with flutamide secreted 81,871 ± 4098 pg/mL of IL-6. The highest IL-6 suppression effect was evident in cells treated with E2. Treatment of stimulated RAW264.7 cell culture with E2 suppressed IL-6 to 13,329 ± 1971 pg/mL. Tamoxifen or flutamide alone had no suppression effect on IL-6 secretion in stimulated RAW264.7 cell culture (data not shown). Addition of tamoxifen with an antagonistic effect significantly reversed the anti-inflammatory effects of E2. The combination of E2 and tamoxifen secreted 32,871 ± 3093 pg/mL of IL-6. A relatively low suppression effect on IL-6 secretion was induced by BPA treatment. BPA alone induced 62,671 ± 2903 pg/mL of IL-6, which was reversed when combined with tamoxifen to 102,529 ± 3235 pg/mL of IL-6.

## 4. Discussion

Pollutants with estrogenic and androgenic effects are common in wastewater treatment facilities and receiving water bodies [4,35]. As a result, there have been increasing concerns over their association with several adverse effects to the environment, animals, and humans [3]. The most common adverse health effect reported is endocrine disruption, especially reproductive system modulation [36]. Apart from the disruption of the reproductive system, an increasing number of reports suggest that androgenic and estrogenic compounds can also cause immune disruption. The disruptions of immune functions occur in many different cell types of the immune system, including macrophages [9,10,11,12,19]. Based on the possible effects of estrogenic and androgenic compounds on the immune system, the present study evaluated whether exposure of macrophages to common EDCs such as DHT, E2, and BPA modulate inflammatory biomarker production. These EDCs were selected for investigation due to literature available showing that they can be found in most water bodies impacted by human activity [6,37]. The study used a murine macrophage RAW264.7 cell line as a model for inflammatory responses. The inhibition of inflammation was assessed using NO and IL-6 as biomarkers of an inflammatory response. The results show that 5 μg/mL of DHT, E2, and BPA had no negative effect on RAW264.7 cell viability and were therefore not toxic.

The results further show that exposure of LPS-stimulated RAW264.7 cells to 5 μg/mL of DHT, E2, or BPA suppressed inflammatory biomarker secretion. The inhibition of inflammatory effects ischaracterized by significant decreases of NO and IL-6 secretions, compared with the positive control stimulated with 1 μg/mL LPS. These results indicate that DHT, E2, and BPA suppressed both NO and IL-6 secretion in stimulated RAW264.7 cells.

The actions of DHT through androgen receptors (ARs) are similar to those of testosterone, because DHT is a more biologically active form of testosterone. DHT is a derivative of testosterone synthesized from testosterone by enzymatic action of 5-reductase [7,8]. Its actions are also through ARs, whose expression has been detected in various immune cell types, such as macrophages [19,20]. The involvement of AR in the anti-inflammatory effects of DHT was confirmed by the results of combined DHT and flutamide treatment. The combined treatment of flutamide with DHT reversed the inhibition of inflammatory biomarker secretion by RAW264.7 cells. This is probably because flutamide is a selective antagonist of the AR [38]. A similar inhibition response in macrophage RAW264.7 cells culture might be induced by environmental androgenic pollutants. This is because androgens are common in sewage effluents and surface water bodies [6,37]. Therefore, these results suggest that anti-inflammatory activities of androgens, which are common in wastewater, can be assessed by the use of stimulated RAW264.7 cells culture. However, molecular mechanisms of androgen action on AR in macrophages require further studies.

Similarly, inhibitions of inflammatory biomarkers were evident upon treatment of stimulated RAW264.7 cells with E2 and BPA. The treatment of stimulated RAW264.7 cells with E2 and BPA suppressed inflammatory biomarkers by decreasing secretion of both NO and IL-6. The effects were more evident with E2 than BPA treatment. Both E2 and BPA can bind to estrogenic receptors (ERs), which are expressed in many immune cells including macrophages. The difference of E2 and BPA effects could be associated with differences in their affinity to ERs [39,40]. E2 is normally characterized by stronger estrogenic activities compared to BPA, which is characterized by weaker estrogenic activities [3]. Involvement of ERs in E2 and BPA inhibition of inflammatory biomarkers was confirmed by the results where LPS-activated RAW264.7 cells were exposed to combinations of E2 and BPA with tamoxifen. Tamoxifen is an ER antagonist [41]. Likewise, anti-inflammatory activities of estrogen might be induced by estrogenic pollutants. Estrogenic hormones have been frequently detected in sewage effluents and surface water bodies [35,37]. Hence, the findings of the present study suggest that anti-inflammatory activities of estrogenic pollutants can be evaluated by using stimulated RWA264.7 cell culture. Nevertheless, molecular mechanisms involving ER in macrophages may require further studies.

## 5. Conclusions

The results of the present study show that DHT, E2, and BPA, which are common EDCs in wastewater, can disrupt inflammatory response and hence immune function. The results show further that DHT, E2, and BPA can inhibit inflammatory mediator production in macrophages, partly through their respective steroid hormone receptors. These findings show that stimulated RAW264.7 macrophages may be a useful model for evaluation of inflammatory mediator inhibition activities of estrogenic and androgenic contaminants that are common in wastewater.
